# Seroprevalence and correlates of herpes simplex virus type 2 infection among young adults in Arkhangelsk, Northwest Russia: a population-based cross-sectional study

**DOI:** 10.1186/s12879-016-1954-8

**Published:** 2016-10-28

**Authors:** Tatiana Balaeva, Andrej M. Grjibovski, Oleg Sidorenkov, Olga Samodova, Natalia Firsova, Anatoly Sannikov, Elise Klouman

**Affiliations:** 1 Department of Community Medicine, UiT The Arctic University of Norway, PO Box 6050 Langnes, N-9037 Tromso, Norway; 2 Northern State Medical University, Troitski Av. 51, Arkhangelsk, 163000 Russia; 3 Center of Hygiene and Epidemiology in the Arkhangelsk Region, Troitski Av. 164-1, Arkhangelsk, 163000 Russia; 4 North-Eastern Federal University, 58 Belinsky Str, Yakutsk, 677000 Yakutsk, Republic of Sakha (Yakutia) Russia; 5 Department of International Public Health, Norwegian Institute of Public Health, PO Box 4404 Nydalen, N-0403 Oslo, Norway; 6 Department of Preventive Medicine, International Kazakh-Turkish University, Turkestan, Kazakhstan; 7 Arkhangelsk Regional Dermatovenerologic Dispensary, Sibiryakovtsev Street 2-1, Arkhangelsk, 163045 Russia

**Keywords:** Herpes simplex virus type 2, HSV-2, Correlates of HSV-2, Self-reported STIs, Seroprevalence, Population-based, Northwest Russia, Eastern Europe

## Abstract

**Background:**

Herpes simplex virus type 2 (HSV-2) infection is the most common cause of genital ulcer disease (GUD) worldwide. Mother to child transmission causes high morbidity and mortality among infants. Russia is on the brink of a generalized HIV-epidemic, but Arkhangelsk is still a low-prevalence area. HSV-2 infection is associated with a three-fold increased risk of HIV-infection. The evidence on the seroprevalence of HSV-2 in Russia is limited. The aim of this study was to assess HSV-2 seroprevalence and correlates among young adults in the city of Arkhangelsk.

**Methods:**

1243 adults aged 18-39 years participated in a cross-sectional population-based study, recruited by a public opinion agency applying a quota sampling method to achieve a data set with similar age- and sex-distribution as the population in Arkhangelsk. All participants completed a standardized, self-administrated questionnaire and were tested for HSV-2. Associations between HSV-2 seropositivity and selected sociodemographic and behavioral factors, and self-reported history of sexually transmitted infections (STIs) were studied by multivariable logistic regression.

**Results:**

HSV-2 seroprevalence was 18.8 %: 12.2 % (95 % confidence interval, CI 9.7-15.2) among men and 24.0 % (95 % CI 20.1-27.3) among women. Among men, HSV-2 positivity was associated with being divorced/widowed (OR = 2.85, 95 % CI 1.06-7.70), cohabitation (OR = 2.45, 95 % CI 1.07-5.62), and a history of STIs (OR = 2.11, 95 % CI 1.14-3.91). In women, HSV-2 positivity was associated with high income (OR = 3.11, 95 % CI 1.45-6.71) and having a lifetime number of sexual partners between 2 and 5 (OR = 2.72, 95 % CI 1.14-6.51), whereas sexual debut at age 18 years or older was inversely associated with the outcome (OR = 0.47, 95 % CI 0.31-0.72). In both sexes, increasing age was the strongest correlate of HSV-2 seropositivity in multivariable analyses.

**Conclusion:**

The HSV-2 seroprevalence was twice as high in women than in men and increased with age in both sexes, and similar to that reported from high-prevalence countries in Europe and the USA. The high prevalence of HSV-2 among women in childbearing age reveals the potential for HSV-2 transmission from mothers to infants and increased risk of acquisition HIV-infection; it also contributes to the burden GUD among both sexes. This emphasizes the public health implications of the HSV-2 epidemic in an urban population in North-West Russia.

## Background

Genital herpes simplex virus type 2 (HSV-2) is one of the most common sexually transmitted infections (STIs) worldwide, affecting one in every 10 individuals [1]. HSV-2 goes through periods of non-replication (i.e., latency), and periods of reactivation, during which viral shedding occurs from the site of initial infection. HSV-2 infection is lifelong, and there is no cure. Most infected persons are not aware that they carry the virus, but both asymptomatic and symptomatic persons can transmit the virus to others. HSV-2 is the dominant cause of genital ulcer disease worldwide and can cause disseminated infection and central nervous system disease complications. Moreover, transmission of HSV-2 from mother to child during pregnancy causes high morbidity and mortality among infected infants [1–3]. Most infections among infants are transmitted during delivery, and more efficiently transmitted in primary infection near term than during a long-standing HSV-2 infection [4].

Generally speaking, the more sexual partners people have in their lifetime, the greater their risk of HSV-2 infection, making HSV-2 seropositivity a marker for high-risk sexual behavior. Nevertheless, HSV-2 seroprevalence is usually higher in women than men in both high- and low-risk groups [2, 5, 6]. HSV-2 infection is also an important co-factor for HIV infection [7, 8], associated with a three-fold increased risk of HIV infection among both men and women [7].

Routine screening of the general population for HSV-2 is not recommended, and it is impossible to estimate the prevalence of HSV-2 infection among the general population based on clinical case-reporting. Thus, population-based seroepidemiological studies must be carried out to determine the burden of HSV-2 infection [5]. The prevalence of HSV-2 infection varies markedly not only between, but also within countries and population subgroups [6]. Previous studies have shown that HSV-2 seroprevalence is highest in Africa and South America, lower in Western and Southern Europe than in Northern Europe and North America, and lowest in Asia [5, 6]. However, to our knowledge, only one study has been published on HSV-2 seroprevalence in the general population of Russia in a letter to the editor [9]. An overall seroprevalence of 20.3 % was reported in a mixed ethnic population in Siberia, aged 25 to 64 years; 25.3 % in women and 15.5 % in men.

UNAIDS estimated the HIV-prevalence among the general population in Russia in the age-group 15-49 years to be about 1.1 %, i.e., on the brink of a generalized epidemic at the time of the study [10]. Based on case-reporting, the comparable HIV-prevalence in Arkhangelsk region was 0,04 % in 2011 among the general population, rising to 0,08 % in 2015 and on the national level to 0,9 % [11–13]. This underscores that Arkhangelsk region remains a low-prevalence area for HIV-infection; 63.9 % of cases were reported as heterosexual and 28.5 % as parenteral transmission in the period 1992-2015 [11, 13].

Russia is a multiethnic country with a huge amount of territory and different levels of economic and social development. Northwest Russia has a border with the European Union, regular international contacts, and different cross-border activities. Travel, culture, socioeconomic factors and mixing of different population groups contribute to the spread of STIs. Studies on the seroprevalence of HSV-2 may help us to understand better the development of the Russian HIV epidemic, as HSV-2 infection is an important co-factor that contributes to the spread of HIV in the country. Thus, the aim of this study was to assess HSV-2 seroprevalence and its correlates among young adults in the city of Arkhangelsk, Northwest Russia.

## Methods

This population-based cross-sectional study was designed as a “second-generation HIV/STI survey”, which combines biological indicators with data on risk behavior to provide information for HIV/STI preventive efforts [14]. First, a pilot study was carried out with 94 participants. Following the pilot study, the wording of the questionnaire was improved and a new recruitment procedure established. The sample size of 1042 was required to detect odds ratios of two or greater in multiple logistic regression analysis with up to 7 independent variables for the prevalence of the outcome of 5 % or higher with 95 % confidence level. We aimed at recruiting 1200 subjects taking into account potential withdrawals and missing data.

### Study population and enrollment

**Fig. 1:**
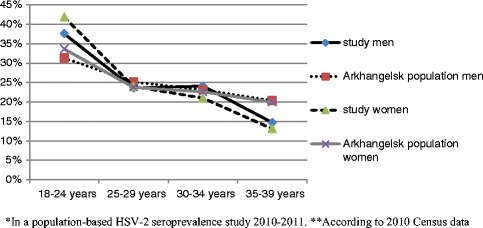
Age and sex among participants * and in the general population**, Arkhangelsk, Russia

We chose to study young adults because they are sexually active and at risk for STIs. Census data could not be used to select a random sample of the population of Arkhangelsk, as individual-level census data on date of birth and sex were not available for research purposes. Instead, participants were recruited by a public opinion agency applying a quota sampling method to achieve a data set similar to the population in Arkhangelsk [15]. The agency had a database of all cellphone numbers in the city, as well as population-based group-level census data on sex and age distribution (“quotas”) in the eight districts of Arkhangelsk. In Arkhangelsk region, the number of cellphones was high in 2011 (1890/1000 population, according to information from Territorial body of Federal State Statistics Service of the Arkhangelsk region). Inhabitants were contacted on their cellphones by the agency, and eligible persons aged 18-39 years were informed about the aims of the study using a script provided by the study team. The invitees who agreed to participate presented themselves at the University Clinic of the Northern State Medical University. Compared to the group-level census data from Arkhangelsk, women and younger individuals were slightly overrepresented in our study sample (Fig. 1) (Inserted approximately here). Enrollment went on between September 2010 and June 2011. Altogether, 4872 respondents completed the “quotas” according to gender and age-groups. Of them, 3973 agreed to participate. However, the study team ended inclusion after enrollment of 1265 participants since this sample size was sufficient according to power calculations. Upon arrival at the clinic, invitees were again informed about aims of the study, after which they signed a written informed consent form. Participants then answered a standardized, self-administrated questionnaire and had a blood sample drawn. Participants were paid approximately 14 USD as compensation for travel costs and discomfort during blood sampling. Twenty-two participants had missing blood test results and were excluded from the analyses, resulting in a study sample of 1243 participants.

### Description of the variables

The questionnaire collected information on sociodemographic and socioeconomic factors, sexual and preventive behavior, alcohol use, a history of STIs/HIV, HIV-testing and a history of genital herpes. Participants reported their family income on a scale of 1 to 10, based on how they thought their income compared to the average income in Arkhangelsk. We then categorized these responses as low (1-4), medium (5-7), and high (8-10). Non-smokers comprised the never-smoking group, and occasional, daily, and ex-smokers were categorized as ever-smokers. The variables “ever-use of condoms” and “condom use at sexual debut” were also dichotomized (Tables 1, 2, 3, 4).

**Table 1:** HSV-2 seroprevalence and correlates among 543 men aged 18-39 years by crude logistic regression analysis in a population-based study, Arkhangelsk, Russia, 2010-2011

Variables	N (%)	HSV-2-positive (%)	Crude OR (95 % CI)*
Age			
18-24 years	204 (37.6 %)	7 (3.4 %)	referent
25-29 years	128 (23.6 %)	17 (13.3 %)	**4.31 (1.73-10.71)**
30-34 years	131 (24.1 %)	26 (19.8 %)	**6.97 (2.93-16.59)**
35-39 years	80 (14.7 %)	16 (20.0 %)	**7.04 (2.77-17.87)**
Marrital status			
Single	266 (49.0 %)	16 (6.0 %)	referent
Divorced/widowed	35 (6.4 %)	10 (28.6 %)	**6.25 (2.56-15.23)**
Cohabiting	74 (13.6 %)	15 (20.3 %)	**3.97 (1.86-8.49)**
Married	168 (30.9 %)	25 (14.9 %)	**2.73 (1.41-5.29)**
Education			
Low (secondary school or less)	124 (22.8 %)	23 (18.5 %)	referent
Average (secondary/vocational)	147 (27.1 %)	17 (11.6 %)	0.57 (0.29-1.13)
High (incomplete higher/higher)	272 (40.1 %)	26 (9.6 %)	**0.46 (0.25-0.85)**
Income			
Low	92 (16.9 %)	10 (10.9 %)	referent
Medium	380 (70.0 %)	46 (12.1 %)	1.13 (0.55-2.33)
High	71 (13.1 %)	10 (14.1 %)	1.34 (0.53-3.43)
Smoking status			
Never	151 (27.8 %)	8 (5.3 %)	referent
Ever	392 (72.2 %)	56 (14.3 %)	**3.10 (1.45-6.67)**
Age at sexual debut			
<18 years	370 (68.1 %)	53 (14.3 %)	referent
≥18 years	173 (31.9 %)	13 (7.5 %)	**0.55 (0.39-0.79)**
Ever-use of condoms			
Yes†	530 (97.6 %)	65 (12.3 %)	referent
No	13 (2.4 %)	1 (7.7 %)	0.60 (0.08-4.66)
Condom use at sexual debut			
Yes†	262 (48.3 %)	24 (9.2 %)	referent
No/don’t remember‡	281 (51.7 %)	42 (14.9 %)	**1.74 (1.02-2.97)**
Condom use in a steady relationship			
Always/almost always	108 (19.9 %)	8 (7.4 %)	referent
Sometimes or rarely	245 (45.1 %)	31 (14.5 %)	1.81 (0.80-4.08)
Never	190 (35.0 %)	24 (16.6 %)	2.07 (0.91-4.74)
Lifetime number of sexual partners			
0-1	37 (6.8 %)	3 (8.1 %)	Referent
2-5	94 (17.3 %)	5 (5.3 %)	0.64 (0.14-2.81)
≥6	412 (75.9 %)	58 (14.1 %)	1.86 (0.55-6.24)
History of STI			
No/don’t know	453 (83.4 %)	44 (9.7 %)	Referent
Yes	90 (16.6 %)	22 (24.4 %)	**3.01 (1.70 -5.33)**
Sex with a commercial sex worker			
No	283 (52.1 %)	22 (7.8 %)	Referent
Yes/don’t want to answer§	260 (47.9 %)	44 (16.9 %)	**2.41 (1.41-4.16)**
Frequency of drinking alcohol			
Never, monthly or less	170 (31.3 %)	18 (10.6 %)	referent
2-4 times a month	252 (46.4 %)	30 (11.9 %)	1.14 (0.61-2.12)
≥2 times a week	121 (22.3 %)	18 (14.9 %)	1.48 (0.73-2.97)
Binge drinking**			
Never	69 (12.7 %)	6 (8.7 %)	referent
Ever	474 (87.3)	60 (12.7 %)	1.52 (0.63-3.67)

*Significant association in bold, the statistical significance is at the 0.05 level

†Includes 4 men reporting no sex debut (i.e. without any risk of HSV-2 infection)

‡Includes 24 men who did not remember

§Includes 21 men who did not want to answer

**Drinking 6 or more units in one occasion

*Abbreviations*: *OR* odds ratio, *CI* confidence interval, *STI* sexually transmitted infection

**Table 2:** HSV-2 seroprevalence and correlates among 700 women aged 18-39 years by crude logistic regression analysis in a population-based study, Arkhangelsk, Russia, 2010-2011

Variables	N (%)	HSV-2-positive (%)	Crude OR (95 % CI)*
Age			
18-24 years	293 (41.9 %)	33 (11.3 %)	referent
25-29 years	168 (24.0 %)	46 (27.4 %)	**2.97 (1.81-4.88)**
30-34 years	147 (21.0 %)	51 (34.7 %)	**4.19 (2.55-6.88)**
35-39 years	92 (13.1 %)	38 (41.3 %)	**5.54 (3.20-9.62)**
Marrital status			
Single	305 (43.6 %)	48 (15.7 %)	referent
Divorced/widowed	51 (7.3 %)	18 (35.3 %)	**2.92 (1.52-5.60)**
Cohabiting	139 (19.9 %)	44 (31.7 %)	**2.48 (1.55-3.98)**
Married	205 (29.3 %)	58 (28.3 %)	**2.11 (1.37-3.26)**
Education			
Low (secondary school or less)	85 (12.1 %)	30 (35.3 %)	referent
Average (secondary/vocational)	155 (22.1 %)	46 (29.7 %)	0.77 (0.44-1.36)
High (incomplete higher/higher)	460 (65.8 %)	92 (20.0 %)	**0.46 (0.28-0.76)**
Income			
Low	115 (16.4 %)	25 (21.7 %)	referent
Medium	515 (73.6 %)	119 (23.1 %)	1.08 (0.66-1.76)
High	70 (10 %)	24 (34.3 %)	1.88 (0.97-3.65)
Smoking status			
Never	340 (48.6 %)	60 (17.6 %)	referent
Ever	360 (51.4 %)	108 (30.0 %)	**2.0 (1.40-2.86)**
Age at sexual debut			
<18 years	356 (50.9 %)	104 (29.2 %)	referent
≥18 years	344 (49.1 %)	64 (18.6 %)	**0.49 (0.26-0.92)**
Ever-use of condoms			
Yes†	679 (97.0 %)	159 (23.9 %)	referent
No	21 (3.0 %)	9 (42.9 %)	**2.45 (1.02-5.93)**
Condom use at sexual debut			
Yes†	314 (44.9 %)	60 (19.1 %)	referent
No/don’t remember‡	386 (55.1 %)	108 (38.8 %)	**1.65 (1.15-2.35)**
Condom use in a steady relationship			
Always or almost always	162 (23.1 %)	21 (13.0 %)	referent
Sometimes or rarely	318 (45.4 %)	78 (24.5 %)	**2.18 (1.29-3.69)**
Never	220 (31.4 %)	69 (31.4 %)	**3.07 (1.79-5.26)**
Lifetime number of sexual partners			
0-1	97 (13.9 %)	7 (7.2 %)	referent
2-5	330 (47.1 %)	74 (22.4 %)	**3.72 (1.65-8.37)**
≥6	273 (39.0 %)	87 (31.9 %)	**6.01 (2.68-13.52)**
History of STI			
Never/don’t know	543 (77.6 %)	112 (20.6 %)	referent
Yes	157 (22.4 %)	56 (35.7 %)	**2.13 (1.45-3.14)**
Frequency of drinking alcohol			
Never, monthly or less	317 (45.3 %)	67 (21.1 %)	referent
2-4 times a month	290 (41.4 %)	76 (26.2 %)	1.33 (0.91-1.93)
≥2 times a week	93 (13.3 %)	25 (26.9 %)	1.37 (0.81-2.33)
Binge drinking§			
Never	173 (24.7 %)	31 (17.9 %)	referent
Ever	527 (75.3 %)	137 (26.0 %)	**1.61 (1.04-2.49)**

*Significant association in bold, the statistical significance is at the 0.05 level

†Includes 14 women reporting no sex debut (i.e. without any risk of HSV-2 infection)

‡Includes 40 women who did not remember

§Drinking 6 or more units in one occasion

*Abbreviations*: *OR* odds ratio, *CI* confidence interval, *STI* sexually transmitted infection

**Table 3:** HSV-2 infection and correlates among 543 men aged 18-39 years by multivariable logistic regression analysis in a population-based study, Arkhangelsk, Russia, 2010-2011

Variables	Adjusted OR (95 % CI)*
Age	
18-24	referent
25-29	2.70 (0.98-7.23)
30-34	**3.95 (1.46-10.72)**
35-39	**4.05 (1.38-11.86)**
Marital status	
Single	referent
Divorced/widowed	**2.85 (1.06-7.70)**
Cohabiting	**2.45 (1.07-5.62)**
Married	1.52 (0.73-3.19)
Education	
Low (secondary school or less)	referent
Average (secondary/vocational)	0.57 (0.28-1.19)
High (Incomplete higher/higher)	0.71 (0.36-1.39)
Smoking status	
Never	referent
Ever	2.05 (0.89-4.72)
Age at sexual debut	
<18 years	referent
≥18 years	0.53 (0.26-1.07)
Condom use at sexual debut	
Yes†	referent
No/don’t remember‡	1.07 (0.59-1.96)
History of STI	
No/don’t know	referent
Yes	**2.11 (1.14-3.91)**
Sex with a commercial sex worker	
No	referent
Yes/don’t want to answer§	1.35 (0.73-2.48)

*Significant association in bold

†Includes 4 men reporting no sex debut (i.e. without any risk of HSV-2 infection)

‡Includes 24 men who did not remember

§Includes 21 men who did not want to answer

*Abbreviations*: *OR* odds ratio, *CI* confidence interval, *STI* sexually transmitted infection

**Table 4:** HSV-2 infection and correlates among 700 women aged 18-39 years by multivariable logistic regression analysis in a population-based study, Arkhangelsk, Russia, 2010-2011

Variables	Adjusted OR for women (95 % CI)*
Age	
18-24	referent
25-29	**2.84 (1.61-5.03)**
30-34	**4.36 (2.44-7.79)**
35-39	**6.47 (3.23-12.97)**
Marital status	
Single	referent
Divorced/widowed	1.11 (0.52-2.38)
Cohabiting	1.40 (0.82-2.39)
Married	1.07 (0.64-1.77)
Education	
Low (secondary school or less)	referent
Average (secondary/vocational)	0.71 (0.37-1.34)
High (Incomplete higher/higher)	0.62 (0.35-1.10)
Income	
Low	referent
Medium	1.48 (0.85-2.57)
High	**3.11 (1.45-6.71)**
Smoking status	
Never	referent
Ever	1.53 (0.98-2.37)
Age at sexual debut	
<18 years	referent
≥18 years	**0.47 (0.31-0.72)**
Ever-use of condoms	
Yes†	referent
No	2.92 (0.99-8.62)
Condom use at sexual debut	
Yes†	referent
No/don’t remember‡	0.94 (0.62-1.43)
Condom use in a steady relationship	
Always or almost always	referent
Sometimes or rarely	1.30 (0.72-2.33)
Never	1.38 (0.75-2.56)
Lifetime number of sexual partners	
0-1	referent
2-5	**2.72 (1.14-6.51)**
≥6	**2.54 (1.03-6.30)**
History of STI	
No/don’t know	referent
Yes	1.45 (0.94-2.24)
Binge drinking§	
Never	referent
Ever	1.33 (0.80-2.23)

*Significant association in bold

†Includes 14 women reporting no sex debut (i.e. without any risk of HSV-2 infection)

‡Includes 40 women who did not remember

§Drinking 6 or more units in one occasion

*Abbreviations*: *OR* odds ratio, *CI* confidence interval, *STI* sexually transmitted infection

### Serological testing

Blood samples were centrifuged and subsequently frozen at -20 °C at the Research laboratory of the Northern State Medical University. The laboratory of Arkhangelsk Clinical Dermatovenerologic Dispensary tested blood samples for HSV-2 using an enzyme-linked immunosorbent assay (ELISA) for IgG antibodies against the specific HSV-2 glycoprotein g2, according to the manufacturer’s instructions (Vector-Best, Novosibirsk, Russia). The other Russian population-based study on HSV-2 seroprevalence from Siberia used the same test [9] Vector-Best is the largest manufacturer of reagent test kits in Russia, with a well-established quality control system. However, we are not aware of any international publications on the validity of the test kits used in our study. The instruction manual reports the test to be 100 % sensitive and specific. In real-life, we expect a lower performance of the test. We will discuss our test results, based on published guidelines for the management and laboratory diagnosis of genital herpes [16, 17].

### Statistical analyses

Continuous and categorical variables were analyzed using unpaired *t*-tests and Pearson’s chi-squared tests, respectively; Wilson’s method was applied for the calculation of confidence intervals (CI) for proportions. Logistic regression was used to assess the association between HSV-2 seropositivity and potential risk factors, and crude and adjusted odds ratios (ORs) with 95 % CIs are provided. Variables associated with HSV-2 seropositivity with a p-value <0.05 in crude analyses were included in the multivariable models; and analyses were stratified by sex. The Statistical Package for Social Sciences version 20 (IBM SPSS Inc) was used for all analyses. The direct standardization technique was applied to provide the age-standardized HSV-2 seroprevalence to 2010 Arkhangelsk population, based on the 2010 census.

### Ethical approval and confidentiality

The Ethical Committee of the Northern State Medical University in Arkhangelsk approved the study protocol. (Refer to Declarations). The interview, blood samples, and blood test results were labeled with a unique personal code and analyzed in an anonymous manner.

## Results

**Fig. 2:**
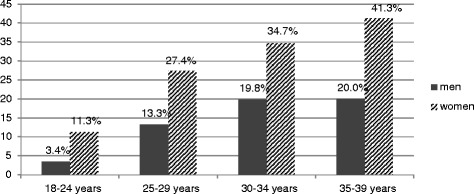
HSV-2 seroprevalence by age and sex in a population-based study, Arkhangelsk, Russia, 2010-2011

Of the 1243 participants, 543 were men and 700 were women (mean age 27.6 years and 27.0 years, respectively). Full-time employment was reported by 64.1 % of men and 48.1 % of women (data not shown), while 40.1 % of men and 65.8 % of women reported high and incomplete high education (Table 1 and 2). Seroprevalence of HSV-2 and characteristics of the study sample are presented in Table 1 and 2. The crude overall HSV-2 seroprevalence was 18.8 % (95 % CI 16.8-21.1) and increased with age in women in all age groups and up to the age of 30 years in men (Fig. 2). (Inserted approximately here) Seroprevalence was higher in women than in men in all age groups and twice as high in overall estimates (24.0 %, 95 % CI 20.1-27.3 among women and 12.2 %, 95 % CI 9.7-15.2 among men; *p* < 0.001) in the sample. After standardization by age, the overall prevalence increased to 20.5 % (95 % CI 18.3-22.7) among the general population; 13.1 % (95 % CI 10.3-15.9) among men and 26.4 % (95 % CI 23.1-29.7) among women.

In crude analyses for men, HSV-2 seropositivity was associated with older age, cohabitation, being divorced/widowed or married, ever-smoking, condom use at sexual debut, a history of STIs, and ever having sex with a commercial sex worker. High education was associated with decreased risk of HSV-2 seropositivity compared with low education (secondary school or less). Men with age at sexual debut <18 years had an HSV-2 seroprevalence that was nearly twice as high as that seen in men who were older at sexual debut (Table 1).

In crude analyses for women, HSV-2 seropositivity was associated with older age, cohabitation, being divorced/widowed or married, ever-smoking, lifetime number of sexual partners, never-use of condoms, no condom use at sexual debut, sometimes/rarely using condoms in a steady relationship, a history of STIs, or binge drinking. Sexual debut at ≥18 years of age was protective. High education was associated with a lower risk of HSV-2 seropositivity. Frequency of drinking was not associated with HSV-2 seropositivity (Table 2).

In adjusted analysis for men, HSV-2 seropositivity was associated with being ≥30 years of age, being divorced/widowed or cohabiting, and having a history of STIs (Table 3). In adjusted analysis for women, HSV-2 seropositivity was positively associated with age, high income, and lifetime number of sexual partners; while sexual debut at ≥18 years of age remained a protective factor (Table 4). Education and smoking in both sexes, and age at sexual debut in men were suggestive of an association with HSV-2 seropositivity although not reaching the level of statistical significance.

Only 18 participants reported no sexual debut, none of whom tested positive for HSV-2. Only 3.6 % of respondents reported a history of genital herpes; more women (4.7 %) than men (2.2 %). In both sexes, one-third of those who reported a history of genital herpes had a negative HSV-2 test. HSV-2 positivity was associated with a history of genital herpes in crude analyses in men (OR = 16.31, 95 % CI 4.76-54.85) and in women (OR = 5.40, 95 % CI 2.62-11.10). On the other hand, 86.8 % of all HSV-2-seropositive participants reported that they had never had genital herpes, i.e. indicating a very high degree of asymptomatic HSV-2 infection; 3 % also reported that they did not know, and only 12 % reported a history of genital herpes (data not shown). Previously HIV testing was reported by 55.4 % of the men and 73.9 % of the women. No one reported being diagnosed with HIV infection. The association between a history of HIV-testing and HSV-2 infection was 2.37 (95 % CI 1.34-4.20) among men and 1.77 (95 % CI 1.15-2.73) among women.

## Discussion

To our knowledge, this is the first study on HSV-2 seroprevalence in an adult population of Northwest Russia. Overall, crude HSV-2 seroprevalence in our study of 18-39-year-olds was 18.8 %: 24.0 % among women and 12.2 % among men. HSV-2 seroprevalence increased with age, more marked in women than in men. This sex and age pattern is common in many populations worldwide, although seroprevalence levels vary greatly [5, 6].

### Seroprevalence of HSV-2 in different Russian subpopulations

We found only one published population-based study in the Russian general population from Siberia reporting an overall seroprevalence of 18.6 % among those aged 25-44 years: 24.1 % in women and 7.5 % and in men [9]. The seroprevalence was lower than observed in our, albeit slightly younger, 25-39-year age group (33.2 % in women and 17.4 % in men). A five-country randomized controlled trial on STI/HIV prevention included 18-30-year-old residents of trade school dormitories in St Petersburg, considered to be at high risk for STI/HIV [18–20]. An exploratory survey carried out in St. Petersburg before the trial showed a seroprevalence of 9.1 % in women and 3.2 % in men [19]. In the baseline study of the trial one year later, an even lower seroprevalence of HSV-2 was found in the much larger study sample (4.0 % in women and 1.4 % in men) [20]. Our study carried out approximately a decade later and included the same age group, revealed a much higher seroprevalence among young adults in Arkhangelsk (17.1 % in women and 7.2 % in men, aged 18-29 years).

A pilot study of 50 male commercial sex workers in Moscow showed a high HIV seroprevalence (16 %), indicating high-risk behavior, but only 4 % of participants were HSV-2-seropositive [21]. Moreover, a study of pregnant women in Israel found that immigrants from the former Soviet Union had a HSV-2 seroprevalence of 27.5 %, i.e., three-fold higher than Israeli-born women. This is comparable to the prevalence we found among women of the same age in Arkhangelsk [22]. Thus our study is in agreement with previous studies of different Russian populations groups which reported a much higher seroprevalence in the general population, than in high-risk groups [9, 18–22]. This pattern is contrary to what is usually found, and underscores the heterogeneity of the HSV-2 seroprevalenc that can be found within one nation.

### Seroprevalence of HSV-2 in other countries

Studies from general population and low-risk populations from Eastern Europe showed an HSV-2 seroprevalence that was either comparable to [23] or lower than [24–26] what we observed. One study showed great variations in seroprevalence in Europe, from 24 % in Bulgaria, 14 % in Germany, 13 % in Finland and 11 % in Belgium, to 9 % in the Netherlands, 6 % in the Czech Republic and 4 % in England and Wales [27]. The seroprevalence we observed in Arkhangelsk coincides with higher-prevalence countries in Europe, and is similar to estimates for Eastern Europe and Central Asia [5]. Our results are also similar to those reported from the USA, where overall HSV-2 seroprevalence was 19 % and women had more than twice the odds of being HSV-2-seropositive compared to men [28].

Our study lies between the extremes of HSV-2 seroprevalence in sub-Saharan Africa, which has the highest seroprevalence in the world (6 %-57 % among men and 29 %-74 % among women) [29], and Asia, which has a lower seroprevalence in most general population groups, i.e. 5 %-7 % among men and 7 %-9 % among women [30–33]. Looker et al estimated a very high seroprevalence among women in Latin America and the Caribbean (38,5 %), identical to the reported seroprevalence among women in Costa Rica, a country in the same region [5, 34]. However, the seroprevalence among 18-35-year-old women in this study was 21 % [34], the same prevalence as in our study women of similar age. Two population-based studies from South America showed a lower HSV-2 seroprevalence than what we observed in Arkhangelsk, and it did not differ between the sexes [35, 36]. These results reveal the great differences in HSV-2 seroprevalence in the general population worldwide. As in Russia, generalization of HSV-2 prevalence to other population groups should be done with caution. However, prevalence studies are the first step in mapping the worldwide spread of this virus.

### Factors associated with HSV-2 infection

In our study, age showed the strongest correlation with HSV-2 seroprevalence in both sexes, which is in agreement with most previous studies and reviews [5, 6, 23–25, 27, 30, 32, 33, 37].

However, in some studies HSV-2 seroprevalence increased in young adulthood and then stabilized [28], as it did with men over 30 years in our study; or decreased at a certain age [9, 35]. Since our oldest participants were only 39 years old, we cannot rule out the possibility that prevalence would increase after that age. HSV-2 seroprevalence among women in our study steadily increased with age. In the Siberian study, prevalence increased with age among men, but decreased in the oldest age group (55-64 years) of women [9]. Thus, comparing prevalence in different age-groups within a specific population, may give an idea of a time trend of the spread HSV-2 within that particular population.

Nearly two thirds of HIV-transmissions are reported to be heterosexual in Arkhangelsk region in the period 1992-2015, and “feminization” of the epidemic is taken place when HIV-infection presently is spreading to the general population in Russia [12, 13]. At this stage of the HIV-epidemic, HSV-2-infected women are of increased risk of acquisition HIV-infection [7]. Even though Arkhangelsk region is a low-prevalence are for HIV- infection, we consider the HIV- prevalence to be somewhat higher for the urban area, Arkhangelsk city, where this study took place. Prevalence based on case-reporting [11–13] will also in general be lower than the real prevalence.

In most studies, including ours, women had higher prevalence of HSV-2 infection than men [5, 6, 19, 20, 23–31, 37], revealing the potential for HSV-2 transmission from mothers to infants. [4]. Low education has consistently been associated with HSV-2 seropositivity [24, 28, 32, 37], and smoking has been reported as a risk factor for HSV-2 infection in some studies [28, 34], but not others [24, 37]. For both sexes, we also found such associations, but it did not reach the level of statistical significance in the multivariable analyses, although close for smoking in women. This might be due to insufficient sample size to detect relatively small effects. Moreover, some studies have reported an inverse association between income and HSV-2 seropositivity [37], whereas other have reported no association [34, 35]. In our study, high income was the strongest predictors of HSV-2 seropositivity among women. Further studies, preferably of qualitative design, needs to clarify this rather unusual finding.

Neither frequency of alcohol drinking nor binge drinking was associated with HSV-2 seropositivity in multivariable analyses in our study. This is in agreement with a study from the USA [28] and in contrast to a study from Japan [30]. Several [28, 29, 32, 34], but not all [24] studies have shown an association between HSV-2 seropositivity and early age at sexual debut. However, these studies are not directly comparable, as the definition of early sexual debut in the studies varied from 13-19 years of age. We found such an association in crude analyses, but only persisting in the multivariable analysis for women. For men, OR was slightly lower in the multivariable, compared to the crude analysis (0.53 vs 0.55), suggesting a protective effect of late sexual debut. However, CI was somewhat wider than in crude analysis, thus not reaching the significant level. This might be due to lack of power in the relatively small sample of men with a lower HSV-2 seroprevalence, compared to women in our study.

Condom use is reported to have a protective effect among women in some studies [31, 34], and the same has been reported for men in Africa [29]. We did see these associations in crude analyses, but they did not persist in multivariable analyses. While diverse results on the protective effect of condom use have been reported, most studies agree that having multiple sexual partners is a risk factor for HSV-2 seropositivity, especially among women as found in our study [22, 24, 29, 31, 32, 37]. Such association was not found among men, who reported far more sexual partners. Different patterns of sexual mixing in women compared to men might expose women to more risk of HSV-2 infection, which adds to a greater biological susceptibility to HSV-2 in women, and may partly explain the higher HSV-2 seroprevalence in women compared to men [33].

The associations between HSV-2 seropositivity and a history of STIs and herpes were investigated in a Romanian study, with results that were similar to those of our study [24]. HSV-1 is increasingly common in genital sites [2], but we did not test for this virus. Thus, the HSV-1 virus may, have caused some of the genital herpes cases reported by HSV-2-negative participants, and some cases may have been tested in the window period before seroconversion that may take up to three months [16]. Finally, our results also demonstrate that HSV-2 infection is usually asymptomatic, a well-documented feature of this infection [2, 5], and of the same magnitude which is estimated in the most recent global estimates of HSV-2 infections [33].

### Limitations and strengths of the study

Due to the cross-sectional design, a temporal relationship between risk behavior and HSV-2 seropositivity could not be established. As we investigated the young adult population, we cannot generalize our results to the whole adult population of Arkhangelsk. In addition, the study questionnaire contained sensitive questions, which may have caused social desirability bias due to underreporting of high-risk behavior. Blood sampling, seen as unpleasant by some, may been a deterrent [38]. One of the potential limitations is the validity of the outcome measurement since there are no international publications on the validity of the test kits produced by Vector-best. The two published European guidelines for the management and laboratory diagnosis of genital herpes, agree that the validity of type specific HSV-2 ELISA tests are high [16, 17]. The East European guidelines refer to sensitivity and specificity of tests that have been evaluated to be approximately 97 % and 98 %, respectively [17]. Applied on our study sample, the HSV-2 prevalence was slightly reduced to 11.6 % for men, 23.1 % for women and overall to 17.7 %.

The main strength of our study is its population-based design, including both sexes. The Arkhangelsk 2010 standardized HSV-2 seroprevalence was slightly higher, but still within the 95 % CI of the crude seroprevalence in our study sample. Considering both age-standardization and a possible limitation of test validity, which slightly correct the measured HSV-2 prevalence in opposite directions, we conclude that the prevalence in our study probably does not differ much from the real HSV-2 prevalence in this age-segment of the population in Arkhangelsk. Even though we could not draw a random sample from the population, we managed to achieve a good representation of younger adults in Arkhangelsk with the sampling method used, which we consider close to what could have been achieved by conventional probability sampling. However, our sampling method cannot rule out selection bias, for instance through recruitment via cellphone holders, therefore generalization of the study results should be avoided. Participants received compensation high enough to pay for a taxi to and from the clinic, and this likely secured participation among people with low income. To our knowledge, this study provides the most updated knowledge of HSV-2 seroprevalence and associated factors among the general population in Russia.

## Conclusion

Associated factors with HSV-2 infection do not differ much from findings in other studies, The HSV-2 seroprevalence was twice as high in women than in men and increased with age in both genders, and similar to that reported from high-prevalence countries in Europe and the USA. The high prevalence of HSV-2 among women in childbearing age reveals the potential for HSV-2 transmission from mothers to infants and increased risk of acquisition HIV-infection; it also contributes to the burden GUD among both sexes. Our findings will be useful to formulate multifaceted strategies for the prevention and control HSV-2 infection in the general population in Northwest Russia. More studies HSV-2 seroprevalence are needed among both low- and high-risk groups in Russia.
